# Exploring the use of experiential learning in promoting the integration of disaster risk reduction into primary school curriculum: A case of Botswana

**DOI:** 10.4102/jamba.v11i1.416

**Published:** 2019-02-21

**Authors:** Sebia Mutasa, Christo Coetzee

**Affiliations:** 1 Botlhale International School, Gaborone, Botswana; 2 Faculty of Environmental Science and Management, North-West University, South Africa

## Abstract

A key imperative of Botswana’s adherence to international, regional and local policy on disaster risk reduction (DRR) relates to creating awareness and an understanding of disaster risk. One avenue of creating an understanding of risk is vested in the integration of DRR into primary school curriculums. Botswana has been slow to adhere to the above-mentioned policy imperatives. This paper argues that the slow pace of integration has been driven by a combination of a lack of clarity on the most appropriate teaching methodology through which to deliver information to young students on a topic as complex as DRR, as well as a general lack of policy and resource support from national government. These assertions are tested in a mixed-method research design that included questionnaires, document reviews and interviews. Questionnaires were administered to 30 teachers drawn from six primary schools in Gaborone, as well as in-depth interviews with two key informants drawn from the Curriculum Development Unit and National Disaster Management Office. The study finds that the experiential learning (EL) method provides an appropriate method by which DRR knowledge can be conveyed within the existing curriculum, as many teachers who have taken the innovative step of integrating DRR into their existing subjects are already implementing key components associated with the EL model. It is also established that although EL provides many potential benefits for an integrated DRR curriculum, the lack of clear policy direction and lack of various supporting resources are preventing the method benefits from being realised for Botswana primary schools.

## Introduction

This article reports on findings on the level of disaster risk reduction^1^ (DRR) knowledge that teachers working in public and private schools in Gaborone have, and how they perceive the integration of DRR into the existing primary school curriculum^2^. The focus of this article will be on the issues listed: teachers’ knowledge of disaster and DRR terminology, policies that guide DRR integration into the primary school curriculum, the importance of teaching DRR themes and topics, challenges in teaching DRR education and how to improve DRR teaching. This article was driven by a review of the Botswana Government’s reporting on progress on Hyogo Framework for Action (HFA) indicators relating to the integration of DRR into formal and non-formal education. This is followed up with questionnaires with teachers and interviews with key informants from the government to ascertain the level of teachers’ DRR knowledge and their impressions as to current curriculum integration efforts. The article concludes by looking at not only some of the stumbling blocks to teachers attaining DRR knowledge and challenges in integrating DRR into the primary school curriculum but also opportunities to improve the current level of DRR knowledge and curriculum integration as they emerged out of the questionnaires and interviews.

Disasters have historically had a massive impact on all sectors of society. Disasters cause losses in life, damage to critical infrastructure and delays in crucial services. These impacts can also be observed in the education sector, with a countless number of lives lost and children’s education postponed indefinitely. Recently, arguments have emerged that state that integrating DRR education in primary school curriculums could limit some of the disastrous effects on children and their immediate community. Some of the benefits of DRR curriculum integration as expounded by Barakat et al. (2010:21), Shaw (2012:232), Tunner et al. (2009:57); Venton and Venton (2012:5) and Wisner (2006:23) include minimising death, injuries and vulnerability to disaster risk throughout society. These authors argue integration of DRR in education curriculum allows for better preparedness and increased capacity and knowledge regarding how to respond in emergency situations as well as helping children to avoid, limit and prepare for the adverse impacts of natural hazards. It is also argued that through the teaching of DRR education, children will have a greater sense of confidence and security as they will be aware of activities that contribute to a reduced psychosocial impact of disasters (Bild & Ibrahim 2013:14). Children are seen as very important information disseminators, as whatever hazard information they learn in school will likely be shared with everyone in the family, thereby increasing the community resilience.

Apart from theoretical arguments calling for and illustrating the benefits of integrating DRR into education curriculum, there is also a concurrent international policy drive to ensure that this integration happens. Within this context, an international disaster risk management policy such as the now lapsed HFA 2005–2015 and the Sendai Framework for Disaster Risk Reduction 2015–2030 have advocated for signatory countries to integrate DRR into national education policies, programmes, and curriculums. Disaster risk reduction integration into education curriculum it is argued will contribute to enhancing community preparedness and vulnerability reduction. Best practice case studies on DRR/Education integration have emerged from all over the globe. In this instance, countries like Japan, Madagascar, Georgia and Cuba have made great strides towards integrating DRR into existing education curriculums. International Federation of the Red Cross – IFRC (2011:44) and Selby and Kagawa (2012:138) confirm that a number of countries worldwide have successfully integrated DRR into school curriculums and have given priority to teacher training in DRR education, amongst these are countries like Madagascar and Angola in Southern Africa; Georgia, Turkey and Russia in Europe; Nepal, Cambodia and Bangladesh in Asia; and Cuba and Peru in Latin America and the Caribbean. However, these success stories in integration between DRR and education curriculums are not the norm, especially within countries in the Southern African Region. Botswana is one such country that still faces challenges towards successful DRR curriculum integration and teacher professional development in relation to DRR teaching.

Botswana is a landlocked country whose hazard profile includes both human-induced and natural hazards (Morof et al. 2013:645). Major hazards that affect both the young and old include drought, floods, wildfires, human immunodeficiency virus (HIV) and acquired immune deficiency syndrome (AIDS), cholera outbreaks, road accidents and animal diseases (National Disaster Management Office [NDMO] 2015:10). Malaria risk is high and covers 80% of the area along the Botswana–Zambia–Zimbabwe border including Chobe, Ngamiland and Okavango districts (Chihanga et al. 2013:8). To address the disaster risk within its borders, the Botswana government has formulated a National Disaster Risk Reduction Strategy (2013–2018). A crucial component of this strategy is its emphasis on government departments to integrate DRR into their main policy, projects and programmes. The implication of this directive for the Ministry of Education and Skills Development is that it should make an effort to integrate DRR into primary and secondary school curriculums.

Naturally, to support DRR integration efforts the Ministry of Education and Skills Development should be cognisant of learning theories that would best support integration efforts. One such theory is embodied by experiential learning (EL) theory. This theory and its utility in integrating DRR into education curriculums will now be discussed.

## Experiential learning: A pathway to integrating disaster risk reduction into Botswana’s primary school curriculum

A plethora of learning theories exist that can be used to build children’s knowledge of and capacity in DRR. These include behaviourist theories, cognitive psychology, constructivism, EL and situated learning theories (Ord 2012:56). Experiential learning is considered to be one of the dominant theories in DRR education because of its utility in fostering integration in teacher professional development as well as DRR student learning. It was therefore used as a guiding theory for this article. Lewis and William (1994:5) and Sharlanova (2004:36) defined EL as learning from experience or learning by doing, this is supported by Kolb (1984:38) who defines EL as ‘a process whereby knowledge is created through the transformation of experience’. Ord (2012) elaborates that EL is based on three key tenets, which are the following:

- People learn best when they are personally involved in the learning experience.
- Knowledge has to be discovered by the individual if it is to have any significant meaning to them or make a difference in their behaviour.
- A person’s commitment to learning is highest when they are free to set their own learning objective and are able to actively pursue them within given frameworks. (p. 55)

**FIGURE 1:**
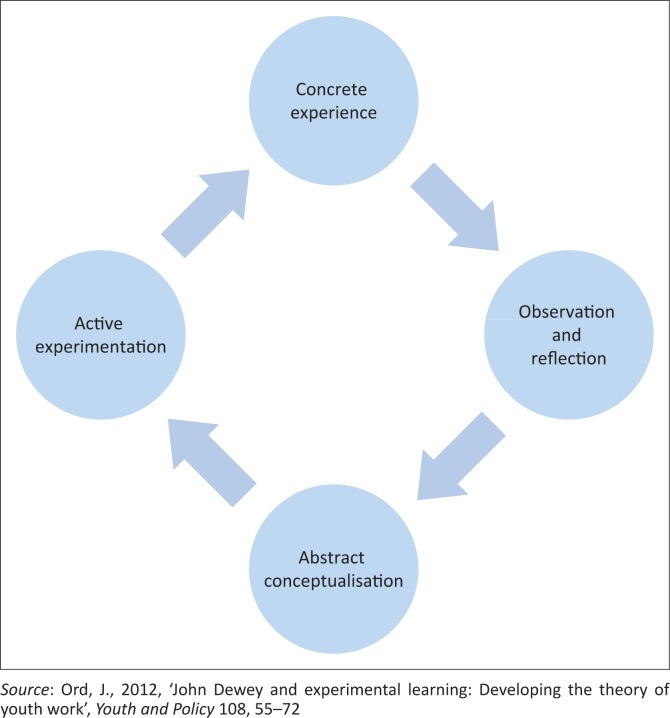
An explanation of Lewin’s experiential learning model.

These assumptions are better explained using the EL model (see Figure 1). The model builds a learning experience that takes students through a four-stage cycle of learning (Kolb 1984:39). The model helps teachers design lessons that are experiential, interactive and that develop critical thinkers. Moon (2004:76) and Risner (2001:5) caution that the four stages in the cycle of learning are often time-consuming and resource-intensive. The four-stage learning cycle involves concrete experience, observation and reflection, abstract conceptualisation and active experimentation whose elements could aid in the development of the DRR-integrated curriculum (Kolb 1984:39; Sharpe 2008:26). Lewis and William (1994:18) and Sharlanova (2004:38) support the EL learning cycle by indicating that it represents the optimal order in which learning takes place. The cycle can be entered at any point and should be seen as a continuously evolving spiral (De Mers 2010:6; Gentry 1990:10). McLeod (2010:12) also views the EL learning cycle as a positive approach to support effective learning as it allows for four steps to take place. These are the following:

- The learner has concrete experience.
- The learner reflects upon their new experience.
- The learner analysis their reflection, observations and create their own conclusion.
- The learner uses these conclusions to test future situations.

After the fourth step, the process repeats itself for every new experience.

Lewin’s EL model, specifically its subcomponents, will be unpacked in the discussion that follows.

One of the primary components of the EL model is concrete experience. Concrete experience is when students and teachers grasp an experience which the student then uses to manage unforeseeable events. It focuses on tangible elements of the immediate environment (Paton & Jackson 2002:76; Sharlanova 2004:38). When students and teachers get together to share hazard knowledge in their environment and the effects of the disaster, it helps bring them closer to reality. The tangible events then become the basis for reflective observation.

Reflective observation is an important process that helps students and teachers to describe a situation objectively and come to an understanding of why things happen (Kolb & Kolb 2005:200). During that process of reflection, students and teachers have to be impartial in order to see the implications and connections and to appreciate different points of view and look for the meaning of things (Lewis & William 1994:16). Reflective observation relies on internal processing, which gives rise to conceptual interpretation which is termed abstract conceptualisation (McLeod 2010:23).

Abstract conceptualisation relies on conceptual interpretation and symbolic representation of the experience (Sharlanova 2004:36). Students and teachers build simple theories from their understanding of concepts such as hazard knowledge, or what causes a volcano, this will then guide their future actions. Abstract conceptualisation can only work well if they are to immediately transform the information into action which then becomes active experimentation.

Active experimentation actively tests the implication of concepts in the new situation to serve as a guide in creating new experiences (Gentry 1990:15; Kolb & Kolb 2005:201). By using this theory, students will be able to apply the learned experience to real-life situations. When students and teachers share their knowledge of hazards and disasters with friends, this empowers them and helps to save lives when a disaster happens because everyone knows what to do.

Alam and Collins (2010:57) and Cox, Calder and Fien (2010:5) argues that ‘experiential learning is important in disaster risk management as learning results from felt or close range interpretation of disaster and development crisis’. Experiential learning situates experience at the core of the learning process and has the potential to motivate participants (teachers and students) to action (Kolb 1984:39; Shaw 2012:239). The benefits of using EL theory in DRR learning is elaborated on in the section that follows.

### Benefits of using experiential learning theory in disaster risk reduction learning

Several studies point to the usefulness of teachers using EL in DRR education. These studies include a study of 400 students in New Zealand who were involved in an EL-based hazard education programme. At the end of the programme, evidence showed that the students demonstrated more stable risk perception, reduced disaster-related fears and a much greater awareness of important hazards (Paton & Jackson 2002:1012). Additionally, they displayed relative protective behaviour as compared to the students who had not experienced hazard education programmes (Carlino, Somma & Mayberry 2008:230). It can be argued that the relative improvement in these students DRR knowledge could only be achieved because of the existence of policies to guide the integration and knowledgeable teachers to facilitate the lessons. Teachers as the custodians of the curriculum need up-skilling for better delivery of DRR teaching. For DRR curriculum integration to be successful, the provision of training and guidance, as well as the availability of student books and teaching guides, is of paramount importance (Selby & Kagawa 2012:40). This reality also provided one of the rationales for using EL as the theoretical base for this paper. For instance, when teachers are supported in acquiring DRR knowledge through experiential professional development workshops, they are able to infuse DRR in every subject they teach. Consequently, they will be able to actively participate in teaching children how to identify and deal with hazards in their own environment without any hindrance. Yamori (2009:91) concur that a curriculum that uses EL approach provides knowledge about hazards as well as involving students in identifying the hazards especially in their local environment (Doud, Cohen & Walker 1983:20). For example, students in Maun district Botswana, which is prone to malaria could theorise in class about malaria and then go into the local environment to identify breeding areas for mosquitoes that exacerbate the malaria problem. It is argued that EL approach is important in DRR education as it links what the students do in class, with what they do at home and in their community. Hence, EL is a perfect approach to teacher professional development as well as inculcating students with knowledge about disaster risk and DRR.

As alluded to earlier in this section, although EL is an excellent vehicle for the integration of DRR into school curriculums, much of its efficacy would depend on the presence of government policies that create a favourable environment for it to be implemented.

### Policies that guide disaster risk reduction curriculum integration

On a policy level, the drive towards DRR integration into the primary school curriculums can be seen by the proliferation of DRR policies, strategies and frameworks that have been produced on multinational and national levels on the issue (Shaw 2012). These include the International Decade for Natural Disaster Reduction 1990–1999, which first mentions the commitment of the international DRR fraternity to push for the integration of DRR principles into education curriculum across the globe. This initial commitment became codified through the HFA (2005–2015) that had the overarching aim of building the resilience of nations and communities to disasters. Specifically, Priority for Action 3 of the HFA states that countries must use knowledge, innovation and education to build a culture of safety and resilience at all levels of society. This, priority for action, therefore, allowed for a breadth of formal and informal education measures, including integrating DRR into the primary school curriculum. At its core, the priority suggested that there could be a substantial reduction in hazards if people are informed about the measures they can use to reduce vulnerability (Tuladhar et al. 2015:21).

In spite of the policy direction provided by the HFA, by the time it was replaced by the Sendai Framework for Disaster Risk Reduction in 2015, evaluations of the progress on achieving Priority 3 indicated slow progress by many nations, especially those in Southern Africa (including Botswana). This lack of progress has been attributed to a limited technical and institutional capacity to formulate fully integrated DRR and education policy at national levels. This lack of integrated policy is augmented with the lack of human and physical resources to practically roll out such policies at subnational levels. The paper finds that some of these issues can also be observed in Botswana’s progress to integrating DRR into its primary school curriculum. However, before these issues can be elucidated, it would be important to highlight the current policy that drives DRR integration into the primary school curriculum in Botswana.

### Status quo of disaster risk reduction education and teacher training in Botswana

Botswana’s National Disaster Risk Reduction Strategy 2013–2018 touches on increasing awareness and knowledge of DRR methods and opportunities and contributing towards the inclusion of DRR into policy, projects and programmes (UNDP & NDMO 2013:9). In line with the HFA priority 3, the strategy attempts to promote awareness of disaster risk in schools and communities (UNDP & NDMO 2013:22). The policy recognises that the integration of risk reduction into the school curriculum may bring the skills and awareness that children need to be able to cope better in disaster situations. In response to the National Disaster Risk Reduction Strategy mandate, the Ministry of Education and Skills Development has started to include information on certain hazards into Botswana primary school curriculum. The inclusion of certain hazards has been done through the infusion of such topics as personal hygiene, HIV/AIDS and safety around the school in lower primary school and safety emphasising on hazards around the school (lightning and traffic) and diseases such as HIV/AIDS in upper primary school. The carrier subjects of these topics are Environmental Science, Creative and Performing Arts, and Social Studies. Although this tentative first step should be commended, the curriculum fails to address significant natural hazards such as drought, floods, animal diseases and wildfires that form a major part of the country’s disaster risk profile. It also places a greater emphasis on hazard awareness than risk reduction per se. These shortcomings are indicative of slow progress in integrating DRR into the school curriculum in Botswana.

This slow progress in integration is confirmed in the country’s reporting on meeting HFA targets. Specifically, progress reports on the implementation of the HFA have revealed that there was very little progress in integrating DRR into the education curriculum. Progress reports submitted for the cycles 2009–2011 and 2013–2015 on the priority area 3 (indicator 2), which relates to the inclusion of DRR knowledge in relevant sections of the school curriculum at all levels showed that Botswana’s national primary school curriculum had not formerly integrated DRR into the education curriculum, in spite of the DRR Strategy recognising that such action is required. The reports also recognise that DRR and the integration thereof in the education curriculums is a fairly new concept to Botswana and much needs to be done in terms of institutionalisation and human and financial resources allocations to realise the goal of integrating DRR into the education curriculums (UNISDR & AU 2013:90). Whilst these shortcomings persist, both teachers and students remain without knowledge on DRR that can contribute to safer communities (Sinkamba & Maripe 2014:5; UNISDR 2014:8).

Botswana attributed its non-compliance with the now lapsed HFA priority 3 to insufficient capacity and resources at NDMO (2015:6). This may indicate why there are no available skills in Botswana to drive the DRR curriculum integration process (NDMO 2015:6).

Regardless of Botswana having such a hazard profile and literature indicating strides that other countries have achieved in integrating DRR into the curriculum and teacher professional development, there is the slow progress of Botswana with implementing DRR integration into the curriculum. In order to understand the reason of the slow progress in Botswana, this paper will aim to ground truth regarding the literature and theory by exploring the overall DRR knowledge of the school teachers and their opinion on DRR integration in the primary school curriculum in Botswana. To this end, questionnaires were administered with the teachers within Gaborone and interviews held with the two government officials who are closely linked to education as it pertains to DRR. The research methods and tools applied during the administration of the questionnaires and interviews are expounded on below.

## Research methodology

A case study research design was applied in the study which involved a mixed-method research approach. A mixed-method research approach as defined by Du Plessis and Majam (2010:456) is a method that involves qualitative and quantitative research methods being mixed in more than one stage of the study. As a point of departure, the research conducted an intensive secondary literature review. Prominent international and national documents, policies and articles were reviewed to get an insight into the theoretical and policy mandates propagating for the integration of DRR in education policies. This review informed the formulation of the research tools used during the questionnaires with teachers and interviews with informants from the government. Specifically, the quantitative research approach allowed the researcher to formulate Likert scale questions, which involved a series of statements that the respondents could choose from in order to rate their degree of agreement or disagreement with the said statement (Teddle & Yu 2007:99; Vogt 1999:336). This quantitative element was supplemented with qualitative open-ended questions that allowed participants to describe their own experiences of the subject in more detail whilst providing the reasoning behind the numbers that came out from the Likert scale questions.

The research tools were applied to a purposefully selected sample of participants that included 30 teachers from 6 primary schools. The primary schools were four government primary schools that follow the government curriculum which provided 12 teachers (three from each school). The four government schools were selected to represent four regions of Gaborone (Gaborone Central Region, Gaborone North-East Region, Gaborone South Region and Gaborone South-East Region). The rationale for selecting schools only in Gaborone was that as the capital of the country, the schools within Gaborone would likely first display the result of any integration of DRR into the education curriculum. Two private schools that follow the government curriculum but enhance the curriculum by adding more flesh to it were also selected and provided three respondents from each school. This was done to give an insight into whether what they teach beyond the government curriculum includes the integration of DRR. Two private schools that offer a curriculum driven by the International Baccalaureate Organisation Curriculum Framework called the Primary Years Programme (PYP) were chosen. This was done to check whether in PYP curriculum framework DRR education is included. The last two were private schools that follow the Cambridge curriculum, some students from these two schools write the Botswana Primary Leaving Exam at grade 7, which follows the government curriculum whilst the other students write Cambridge exam at grade 6. The sample of teachers and schools was selected with the guidance of officials within the Ministry of Education and Skills Development. One link person from the Curriculum Development Unit (CDU) within the Ministry of Education and Skills Development who deals with DRR integration into education curriculum and one person from the NDMO who deals with education and awareness-raising were interviewed. It was not possible to have more than one participant from each of these two departments as the persons interviewed are currently the only persons directly responsible for DRR, and the integration thereof in education curriculum within the respective departments.

Once data were collected from respondents, both quantitative and qualitative data analysis techniques were applied. For the purposes of the qualitative element of research, a narrative analysis was used which is the transcription of experiences and interviews (Teddle & Yu 2007:71). Through the narrative analysis, the researcher had to sort and reflect on the data and enhance and present it in a revised shape to the reader. Coding, which is the process of attaching labels to lines of texts so that the researcher can group and compare similar or related pieces of information, was used (Bless & Higson 2006:102; Greene & Caracelli 1997:10). For the qualitative analysis, a distribution table was created to capture frequency of certain responses emanating from the Likert scale questions (Auriacomb 2010:478; Kendall 2008:45). Some key results from the analysis are conveyed within the section to follow.

## Findings

The following section presents the findings and discussion on the data collected from questionnaires that were advanced to respondents from schools and interviews with the two informants from the key government departments.

### Defining disaster and disaster risk reduction

According to the EL model (see Figure 1), one of the key means of creating an in-depth understanding of a new topic is through the process of abstract conceptualisation. This process allows a person to conceptualise a concept by drawing from their own experiences. As such, one of the questions posed to respondents related to how they would define key concepts such as disaster and DRR. The intention of the question was to gauge the level of understanding of basic DRR concepts amongst teachers, and if this understanding was facilitated through their own personal experiences or through dedicated training and skills development around the topic. This question was directed at the 30 teachers from schools and the majority (28) of them could formulate a definition of the term ‘disasters’. Some of the most often repeated themes in the definitions of the term disaster that was provided by teachers included:

- a terrible situation leading to loss of life and property
- the natural or man-made incident that causes human and environmental damage
- any unexpected incident that causes great destruction.

The majority (20 out of 30) of the teachers could also formulate an informal definition of DRR centred on the following themes:

- minimising or preventing damage caused by disasters
- reducing the rate at which a calamity can take place
- minimising risk before, during and after a disaster.

A comparison of the respondents’ definition and academic definitions of both terms it appeared that the definitions that were provided by respondents were formulated more from teachers own personal understanding and experience of disasters and DRR. In most cases, the definitions provided were more informal in their formulation not using precise terminology that would be indicative of an interaction with a formalised school curriculum that includes scientific definitions of terminology. What was most interesting was both sets of school (private and public) teachers drew from their own experiences, instead of scientifically accurate definitions of disaster and DRR. Scientifically, accurate definitions would have been indicative of some interaction with a curriculum or formal training on the topic of DRR. However, on the plus side, for teachers, abstract conceptualisation drawn from their own experience could indicate the fertile grounds that exist amongst teacher to implement EL-orientated approach to integrating DRR into Botswana’s school curriculum.

### Teaching students about disaster risk reduction

As a follow-up question to the first line of questioning, teachers were asked if they ever taught students about DRR. The majority (20 out of 30) of the teachers indicated that they have taught about DRR, whilst an additional 10 either have never taught (8) about DRR or were not sure (2) if they have touched on topics associated with DRR. Importantly, positive responses were spread out between government and private schools, although teachers in private schools tended to teach about disaster more readily than their state school counterparts. This is in part because some private schools (e.g. PYP schools) already have a unit in the grade 6 syllabus dealing with natural disasters. Interestingly, in the instance of state school teachers that have integrated DRR into some of their subjects have taken the abstract conceptualisation of DRR definitions, and through their own endeavour moved onto what would be known as the active experimentation phase of the EL model. They have thus started to introduce their conceptual understanding of the topic of DRR in the classroom as a means to creating a new learning experience for their students. This unintentional adherence to the basic principles of EL model could be indicative that following EL approach to integrating DRR into the primary school curriculum in Botswana could find fertile ground, as teachers are not afraid to use their own lived experiences to augment their teaching. However, as shown by the results from the private schools involved in the study, the presence of a broad policy and specific subject delineations for integration does increase the likelihood that teachers would teach on the topic of DRR.

### Activities suitable to teaching disaster risk reduction

Teachers were asked what activities they believed would be most suitable to the teaching of DRR. Several examples were highlighted by all participants from all school types (private and public). These included frequent disaster drills, demonstrations, visual learning and visits from DRR experts (fireman, police and disaster management officials) would give children an insight into risks in their immediate environment and possible ways of managing them. These activities would be related to the concrete experience component of the EL model as it would give students insight into the disaster risks in their immediate environment and how to manage them. Furthermore, teachers also indicated that activities such as role play, group discussions, composing songs and poetry about what they observed and practical work would also add value to integrating DRR into the school curriculum. Interestingly, aforementioned activities would all be examples of the reflective observation as per the EL model as it allows students to discuss and explore in greater depth what they observed. Thus, activities that would form part of the concrete experiences and reflective observation components of the EL model are already recognised on an informal level by teachers in Botswana as possible valuable means of integrating knowledge of DRR into the existing curriculum.

## Discussion

The replies from teachers participating in the study point to an underlying implementation of principles associated with EL learning. These principles are being implemented mostly through teachers’ own lived experience of disasters, which in some instance is conveyed to students. This natural inclination to implementing EL principles into existing teaching shows that using an EL approach as a teaching strategy for integrating DRR into Botswana primary school curriculum would not be alien to teachers. This could arguably improve integration efforts as there is already a certain level of familiarity with teaching methods that are consistent with EL. Some responses also indicated that in instances where the innate appreciation for EL methods are supported (in the case of private schools), by policy direction on which subjects and age groups (grades) to focus on, an environment is created whereby teachers are more likely to integrate their experiences of disasters into the curriculum. This can be contrasted with the situation in public schools where a lack of policy direction somewhat inhibits teachers from integrating information about DRR into their lessons. The lack of policy direction is an endemic problem that would have to be addressed in order for EL methods to be utilised in DRR and primary school education in Botswana. Additional lines of questioning that formed part of the larger study that the paper is based also pointed to this. In this regard, teachers and an official from the CDU and NDMO revealed the lack of clear educational policy and institutional objectives that could guide the integration of DRR into primary school curriculums remains the biggest obstacle to integration efforts. In the absence of a clear policy, teachers and officials felt they do not have any direction on the most appropriate subjects and grade levels to integrate DRR concepts into. This ambiguity has probably contributed to teachers’ impression that the integration of DRR into existing curriculums could add work to an already saturated curriculum. Teachers also indicated that the lack of policies and institutional objectives created secondary problems like the absence of teachers’ guides and students’ textbooks and formal training interventions for teachers on the subject of DRR. It can be argued that the lack of policy direction and its associated obstacles are limiting the widespread use of innovative teaching philosophies such as EL, whilst also slowing down progress to integrating DRR into the school curriculum. These problem areas will have to be addressed in order to utilise EL to its full potential and realise the ideal of integrating DRR into the primary school curriculum in Botswana.

## Conclusion

Creating awareness and understanding of disaster risk remains one of the primary goals of global DRR reduction policy and practice. The integration of DRR into school curriculums in general, and primary school curriculums specifically, provides an opportunity to inculcate knowledge about disaster risk and risk reduction from an early age. This integration of DRR into education curriculums could in the long term contribute to generating more disaster-resilient societies. Many countries, including Botswana, are however still struggling to find the appropriate teaching theories and policies that would streamline the integration process. This paper has illustrated that amongst the multitude of learning theories that can be used to stimulate DRR integration into school curriculums, EL provides a compelling option for countries still in the process of integrating DRR into the schooling curriculum. This teaching methodology relies very much on using teachers and student experiences of disaster to build an increasingly complex understanding of disasters and how to reduce their impacts. This study showed that some teachers in Botswana are already implementing some aspects of EL in their teaching of DRR at the primary school level, even though this approach is not directly supported by government policy. This shows that EL seems to be very logical and natural method of teaching a new topic such as DRR for most teachers in the country. This existing affinity with EL methods of teaching could help to streamline future DRR integration efforts into the schooling curriculum. However, it also became clear through the study that policy direction and human, financial and material resources would have to be improved by the Ministry of Education and Skills Development if the potential benefits of EL are ever to be brought to bear in the integration of DRR in the primary school curriculum of Botswana.

## Footnotes

1 Disaster risk reduction is the concept and practice of reducing disaster risk through systematic efforts to analyse and manage the causal factors of disasters, including through reduced exposure to hazards, lessened vulnerability of people and property, wise management of land and the environment and improved preparedness for adverse effects (UNISDR 2011:7)

2 Curriculum is defined by Beauchamp (1977:22) and Su (2012:154) as a means of achieving specific educational goals and objectives.
